# N’Importe

**Published:** 1906-07

**Authors:** 


N'IMPORTE.—*The Texas State Journal of Medicine,* edited by the accomplished Dr. Chase, descends from its dignity and makes an assault upon the *Red Back,* not only totally unwarranted by the facts, but in language unbecoming him. He questions my veracity, and uses such harsh words as "untrue," "garbled," "misleading," "grotesque," etc. To be sure, the charge has no basis in fact, and I feel inclined to pass it up. Every word I said of "The Mixed Board Bill" is literally true. I pass the proffered offense, declining to be offended—it isn't worth while, really. I take to myself the words of Epictetus, the great Stoic philosopher: "Be mild in temper to them that revile you, and remember that it *seems so to them*." In fact, I am mellowed a little by age, I suppose, and am, perhaps, more charitable than I was.

What stung the genial doctor and made him forget his manners was my statement in the May number that "the mixed board bill came to grief and was not even presented to the House of Delegates"; and that the *Red Back's* exposure of the "proposed bill" opened the eyes of the members to the enormity of the thing and killed it. This, with the fact that the "proposed bill" was published exclusively in the *Red Back*, constituting a "scoop on this important piece of news which I (he) would find it difficult to explain" (Dr. Chase's letter to me at the time), seems to have been like a gadfly to his sensitive epidermis. Now, will Dr. Chase say that the "proposed bill" that was sent to the county societies in March for action, and which provided for so many physicians, so many homeopaths, so many eclectics and a pro rata of osteopaths, was even or *ever* presented to the House of Delegates in the form it was submitted to the county societies? Every person present knows that it was not. But, on assembling, Dr. Chase and the others of the committee found such opposition to the mixed character of the bill, an opposition awakened by the *Red Back's* exposure—(for the greater number of those with whom I talked had never seen the thing,—notwithstanding it had been submitted to county societies)—and he stated that the committee deemed it best not to present it in the original form, but to *strike out* the objectionable features (all reference to pathies, the mixed feature). It was accordingly done. The character of the bill was, therefore, entirely changed. The "mixture" was knocked out. Did it not "come to grief"—as stated? It was no longer a bill for a "mixed board," but a bill for *one* board of medical examiners, consisting of *eleven physicians* to be appointed by the Governor. To this bill I made and make no objection, provided the Governor selects the men, and no quack is *asked for* by our grand Association.

The doctor asks why I can not help further the interests of the Association, or words to that effect. It is for that very purpose—furthering the interests and preserving the honor and the dignity and the traditions of legitimate medicine—that I opposed the passage by the House of a bill which would have compromised them all. And it is for the best interests and for the honor and dignity of the regular profession that I have spent the best twenty-odd years of my life in thankless labor.

There seems to be a lower standard held by some of the younger men of today than that established by the fathers.

